# Immunogenic cell death and immunogenic surrender: related but distinct mechanisms of immune surveillance

**DOI:** 10.1038/s41419-021-04178-6

**Published:** 2021-09-24

**Authors:** Rosanna Mezzapelle, Marco E. Bianchi, Massimo P. Crippa

**Affiliations:** 1 Chromatin Dynamics Unit, Division of Genetics and Cell Biology, IRCCS San Raffaele Scientific Institute, Milan, Italy; 2 School of Medicine, Vita-Salute San Raffaele University, Milan, Italy

**Keywords:** Cancer microenvironment, Immunosurveillance

## Introduction

The success of immunotherapies has demonstrated to what extent the immune system can detect, keep in check, and sometimes reverse the development of cancer [1]. Current immunotherapies focus on disabling the PD-1 or CTLA-4 systems, which restrain the activity of cytotoxic T lymphocytes that recognize tumor neoantigens. However, the selection and expansion of such cytotoxic T lymphocyte clones depend on tumor antigen cross-presentation and T cell priming in the first place. If antigens are not detected by the immune system, no immune responses can take place. Dendritic cells sample the microenvironment by phagocytosing exosomes released by living cells or apoptotic bodies deriving from apoptotic cells, and will cross-present new epitopes these contain. Arguably, cells or exosomes bearing neoantigens are not sufficient to elicit a durable immune response; in fact, the same mutational mechanisms generate neoantigens in cancer cells and in aging cells, and yet most aged somatic cells escape the recognition and elimination by the immune system.

If the appearance of neoantigens per se is not the tripwire to kick-start immune responses, what is? This was first answered by the recognition of immunogenic cell death (ICD), a special type of cell death that triggers anti-cancer immune responses against cell neoantigens [2]. Recently, we described “immunogenic surrender”, another mechanism that promotes anti-cancer immune responses [3]. We will argue that ICD and immunogenic surrender are related but distinct biological processes.

## Immunogenic cell death

ICD has been recently defined as “a form of regulated cell death that is sufficient to activate an adaptive immune response in immunocompetent syngeneic hosts” [2]. ICD is characterized by apoptotic morphology with the retention of membrane integrity. Cells that undergo ICD might be targeted by pathogens (including some viruses) or chemotherapeutic agents. The common upstream event in ICD is endoplasmic reticulum (ER) stress with high production of reactive oxygen species (ROS). Some ICD inducers cause ER stress directly, but others cause DNA or chromatin damage, which then initiate ER stress via crosstalk between the DNA damage response (DDR) and the unfolded protein response (UPR) [4]. The characteristic feature of ICD is the activation of the PERK arm of the UPR, which itself is an attempt to react to ER stress: PERK is a kinase (eukaryotic translation-initiation factor 2-alpha kinase 3, EIF2AK3) that is integral to the ER membrane and, as its name implies, phosphorylates and inactivates the alpha subunit of eukaryotic translation-initiation factor 2 (EIF2), leading to a rapid decrease of global protein synthesis [5].

Following UPR activation, cells undergoing ICD release damage-associated molecular patterns (DAMPs) such as calreticulin, heat shock proteins (HSPs), High Mobility Group Box 1 (HMGB1), and ATP (and others as the context dictates). Calreticulin is one of the most abundant proteins in the ER lumen but progresses anterogradely to secretion during ICD; by binding to the surface of the dying cell it signals “eat me” to professional phagocytes. HMGB1 is the archetypal DAMP associated with inflammatory responses and is released late during ICD. HMGB1 has several redox states [6], each of which can bind to several pattern recognition receptors (PRRs) expressed on antigen presenting cells (APCs). Such PRRs include Toll-like receptors (TLR) 2 and 4, which recognize the disulfide form of HMGB1 and activate specific transcriptional and post-transcriptional inflammatory responses. Extracellular ATP functions as a “find-me” signal for professional phagocytes and has additional immunostimulatory effects through inflammasome activation. DNA and RNA molecules released during ICD activate TLR3 and cGAS responses, both in the dying cell and in phagocytes. As a consequence of these multiple and temporally organized stimuli received from the dying cell, professional phagocytes digest fragments of the cell undergoing ICD, and cross-present antigens derived from them to the appropriate cell and in the appropriate location. The ultimate effect is the development of CD8 T cell clones that recognize non-self peptides (either from the pathogen or from tumor-associated antigens) and kill the cells exposing them. Such a complex and finely choreographed interaction between the dying cells and the immune system ensures that non-self antigens are recognized with priority in a context of “danger”, i.e., when a cell is dying in an untimely fashion [7].

## Immunogenic surrender

We discovered immunogenic surrender while testing ICD as a possible mechanism for the anti-cancer therapeutic potential of BoxA, a truncated form of HMGB1 (aa 2–89) endowed with anti-inflammatory properties [8]. Specifically, BoxA extends the survival of immunocompromised mice xenografted with human malignant mesothelioma (MM) cells by interfering with tumor cell proliferation [9]. To test whether BoxA is also effective in immunocompetent hosts, we set up a syngeneic model of MM, where mouse AB1 MM cells (deriving from BALB/c mice) are grafted into the peritoneum of BALB/c mice [10]. Surprisingly, we found that BoxA, besides being anti-proliferative as already shown [9], also promotes protective antitumor immunity, which is responsible for MM rejection and long-term survival in a large fraction of mice [3]. The therapeutic efficacy of BoxA is not limited to MM: immunocompetent mice inoculated with colon carcinoma CT26 cells can also reject them when treated with BoxA.

Because BoxA is a fragment of HMGB1, an actor in ICD, we suspected that BoxA might induce ICD. In fact, BoxA does induce the release of calreticulin and HMGB1; remarkably, though, BoxA does not induce cell death, which by itself excludes ICD as a possible mechanism of anti-cancer immunity. Instead, we found that BoxA binds to the CXCR4 receptor, which is in physical contact with CD47 in resting conditions (i.e., in the absence of ligands). Such binding causes the internalization of CXCR4 and the cointernalization of CD47.

CD47 is a ubiquitous transmembrane protein that prevents the phagocytosis of functionally fit cells by interacting with its ligand SIRPα (signal regulatory protein α) on the surface of macrophages and dendritic cells (DCs) [11]. Lack of CD47 on blood cells results in their rapid clearance by macrophages [12]. CD47 is overexpressed on the cell surface by a variety of malignant cells [13]; its blockade with monoclonal antibodies allows phagocytosis of cancer cells and leads to tumor rejection and development of antitumor immunity [14].

In accordance with surface depletion of CD47, cancer cells exposed to BoxA are readily phagocytosed by macrophages [3]. Interestingly, CD47 internalization and phagocytosis by macrophages are also induced by CXCL12, the canonical ligand of CXCR4. CXCL12 is a homeostatic chemokine and is expressed by a large variety of cells; in fact, the CXCL12-CXCR4 pathway is involved in the regeneration of various damaged tissues, and promotes cell proliferation [15].

## Are ICD and immunogenic surrender different?

At first glance, ICD and immunogenic surrender are two antitumor immune processes that share some of the same molecular actors, most notably the release of the DAMPs calreticulin, ATP, and HMGB1. To what extent are they related or really different?

In ICD apoptotic bodies and corpses are ingested by DCs [2], whereas in immunogenic surrender living cells are ingested by macrophages [3]. However, the difference might not be substantial: we are not aware of experiments testing a role for macrophages in ICD, and a role for DCs in immunogenic surrender.

A second difference is at the level of the UPR. Whereas only the PERK-eIF2α branch of the UPR is activated in ICD, all three are activated in immunogenic surrender. Yet, we did not test if the XBP1 and ATF6 branches are necessary for immunogenic surrender.

To compare ICD and immunogenic surrender, we focus here on the role of DAMPs, and more specifically of extracellular HMGB1. Extracellular HMGB1 released by dying cancer cells and its receptor TLR4 on dendritic cells have been shown to be necessary for ICD [16, 17].

**Fig. 1:**
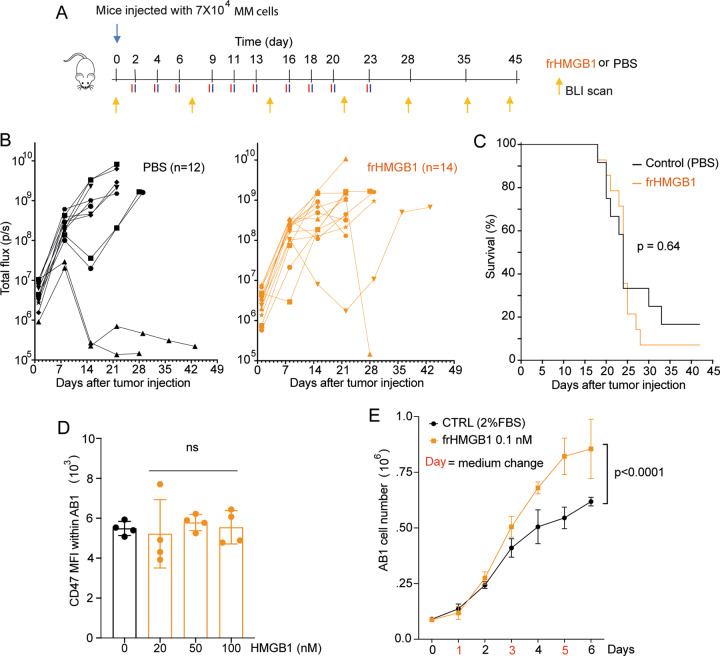
frHMGB1 administration does not confer a survival advantage to mesothelioma bearing mice. **A** Scheme of the in vivo experiment performed and analyzed as described [3]. Briefly: 7 × 10^4^ AB1-B/c-LUC cells were injected i.p. in 6–8 weeks old male BALB/c mice at day 0. Fifty μg of frHMGB1 (HMGBiotech, Milano) or PBS (control) were injected i.p. three times a week and bio-luminescence imaging (BLI) scans were performed once a week. **B** Tumor growth, as monitored by BLI, was similar in control and frHMGB1-treated mice. **C** Survival curves of HMGB1-injected and control mice were not significantly different. Statistics: log-rank Mantel-Cox test. **D** Surface CD47 on MM cells was evaluated by flow cytometry after incubation for 24 h with the indicated concentrations of frHMGB1. The experiment shown is representative of the two performed. Error bars indicate standard deviation. Statistics: one-way ANOVA. ns = not significant. **E** frHMGB1 was added to the culture medium of 10^5^ AB1-B/c-LUC cells seeded in 6-well plates (starting at day 1). Cells were counted every day for 6 days. Statistics: two-way ANOVA.

If extracellular HMGB1 were needed in eliciting antitumor responses in immunogenic surrender, like it is in ICD, injecting it should augment antitumor immune responses. BALB/c mice engrafted with AB1 MM cells were treated or not with 50 μg of fully reduced HMGB1 (frHMGB1) three times a week (Fig. 1A). Untreated and frHMGB1-treated mice showed a similar rapid increase in bio-luminescence imaging signal (BLI, representative of tumor growth; Fig. 1B) and no significant difference in survival (Fig. 1C, *p* = 0.64). Thus, the direct administration of HMGB1 fails to modulate immunogenic surrender in the MM model.

CD47 surface expression is a key determinant of immunogenic surrender. However, HMGB1 did not reduce surface CD47 on AB1 MM cells at all tested doses (Fig. 1D). In contrast, the addition of 0.1 nM frHMGB1 to the medium of MM cells induced faster growth compared to untreated cells (Fig. 1E).

**Fig. 2:**
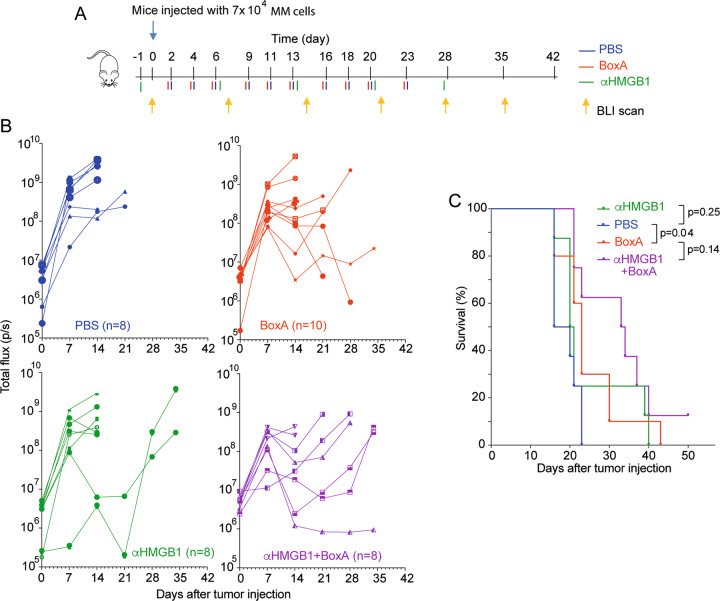
Systemic depletion of HMGB1 does not affect tumor growth in vivo. **A** Scheme of the in vivo experiment (as in Fig. 1) performed and analyzed as described [3]. BoxA (32 mg/kg; HMGBiotech, Milano) or PBS were injected three times per week, anti-HMGB1 antibody (200 μg; HMGBiotech, Milano) once per week. **B** Tumor growth (as monitored by BLI) and **C** survival curves of the four experimental groups. Statistics: log-rank Mantel-Cox test.

We then tested the systemic depletion of extracellular HMGB1. If extracellular HMGB1 were necessary for immunogenic surrender, its blockade would abrogate the therapeutic effects of BoxA. We treated BALB/c mice bearing engrafted AB1 MM cells with PBS, with an anti-HMGB1 neutralizing antibody (clone DPH1.1, 200 μg, once a week), with BoxA (800 μg, three times a week) or both (Fig. 2A). We found that mice receiving anti-HMGB1 antibody alone did not fare worse than control mice; moreover, the concomitant treatment with anti-HMGB1 and BoxA still exerted a therapeutic effect (Fig. 2B, C).

Overall, our results indicate that extracellular HMGB1 does not promote CD47 depletion from the cell surface and that in vivo its blockade or exogenous addition does not alter immunogenic surrender.

## Perspective

Based on the published evidence [3] and on the data we show here, we conclude that ICD and immunogenic surrender differ in at least two respects: first, ICD is a form of regulated cell death, whereas in immunogenic surrender the cells do not die in a cell-autonomous way; second, extracellular HMGB1 is released by tumor cells in both mechanisms, but it is necessary for ICD and not for immunogenic surrender.

We speculate that the first difference, in the mode of cell death as source of antigens, might indicate that ICD and immunogenic surrender are complementary responses of the organism to pathogens. ICD senses cell death, whereas immunogenic surrender senses the abnormal growth of cells. Indeed, many viruses are not cytotoxic but, on the contrary, induce the proliferation of the cells they infect as a way to promote the multiplication of the factories that produce them. Human papilloma viruses (HPVs) are the standard example: they express both an anti-apoptotic protein (E6) and a protein that blocks the host Rb protein thereby activating the cell cycle (E7). Accordingly, several HPVs also induce cancer. Less predictably, protein E6 and E7 promote the secretion of CXCL12, which itself promotes the growth of infected cells via CXCR4 [18]. Another example is human T-cell leukemia virus type 1 (HTLV-1), which induces the overexpression of both CXCR4 and CXCL12, at least in part via the viral protein Tax-1 [19]. Further work, however, is needed to ascertain whether HPVs and HTLV-1 do activate immunogenic surrender.

In this context, the difference in the role of extracellular HMGB1, necessary in ICD and dispensable in immunogenic surrender, supports the notion that the two mechanisms are indeed different. HMGB1 might be released as a consequence of ER stress, although to our knowledge this has not been investigated. On the other hand, our results [3] show that exposure of cancer cells to relatively high levels of CXCL12 induces the UPR and calreticulin release, which together with CD47 internalization contribute to cell phagocytosis by macrophages.

In conclusion, we suggest that immunogenic surrender differs from ICD in the initial trigger: cell proliferation rather than cell death. Both are the exception rather than the rule in adults, and both can offer cues that rouse the attention of the immune system to pathogens, and viruses in particular. CXCL12 induces the UPR, the release of calreticulin and the cointernalization of CD47, which concur in promoting the phagocytosis of living cells by macrophages. Downstream events in ICD and immunogenic surrender, i.e., the cross-presentation of peptides by APCs to CD4 T cells and the expansion of relevant CD8 T clones, might be identical. Immunogenic surrender and ICD may have evolved independently, but both feed signals to the subsequent cellular event: antigen recognition. Thus, it is fortunate but not completely unexpected that both ICD and immunogenic surrender can be exploited to fight cancer.

## Data Availability

The datasets generated during and/or analyzed during the current study are available from the corresponding author on reasonable request.

## References

[1] Ribas A, Wolchok JD (2018). Cancer immunotherapy using checkpoint blockade. Science.

[2] Galluzzi L, Vitale I, Warren S, Adjemian S, Agostinis P, Buqué Martinez A (2020). Consensus guidelines for the definition, detection and interpretation of immunogenic cell death. J Immunother Cancer.

[3] Mezzapelle R, De Marchis F, Passera C, Leo M, Brambilla F, Colombo F (2021). CXCR4 engagement triggers CD47 internalization and antitumor immunization in a mouse model of mesothelioma. EMBO Mol Med.

[4] González-Quiroz M, Blondel A, Sagredo A, Hetz C, Chevet E, Pedeux R (2020). When endoplasmic reticulum proteostasis meets the DNA damage response. Trends Cell Biol.

[5] Bezu L, Sauvat A, Humeau J, Gomes-da-Silva LC, Iribarren K, Forveille S (2018). eIF2α phosphorylation is pathognomonic for immunogenic cell death. Cell Death Differ.

[6] Bianchi ME, Crippa MP, Manfredi AA, Mezzapelle R, Rovere Querini P, Venereau E (2017). High-mobility group box 1 protein orchestrates responses to tissue damage via inflammation, innate and adaptive immunity, and tissue repair. Immunol Rev.

[7] Matzinger P (2002). The danger model: a renewed sense of self. Science.

[8] Venereau E, De Leo F, Mezzapelle R, Careccia G, Musco G, Bianchi ME (2016). HMGB1 as biomarker and drug target. Pharmacol Res.

[9] Yang H, Pellegrini L, Napolitano A, Giorgi C, Jube S, Preti A (2015). Aspirin delays mesothelioma growth by inhibiting HMGB1-mediated tumor progression. Cell Death Dis.

[10] Mezzapelle R, Rrapaj E, Gatti E, Ceriotti C, De Marchis F, Preti A (2016). Human malignant mesothelioma is recapitulated in immunocompetent BALB/c mice injected with murine AB cells. Sci Rep.

[11] Barclay AN, Van Den Berg TK (2014). The interaction between signal regulatory protein alpha (SIRPα) and CD47: structure, function, and therapeutic target. Annu Rev Immunol.

[12] Blazar BR, Lindsberg FP, Ingulli E, Panoskaltsis-Mortari PA, Oldenborg PA, Iizuka K (2001). CD47 (integrin-associated protein) engagement of dendritic cell and macrophage counterreceptors is required to prevent the clearance of donor lymphohematopoietic cells. J Exp Med.

[13] Willingham SB, Volkmer JP, Gentles AJ, Sahoo D, Dalerba P, Mitra SS (2012). The CD47-signal regulatory protein alpha (SIRPα) interaction is a therapeutic target for human solid tumors. Proc Natl Acad Sci USA.

[14] Liu X, Pu Y, Cron K, Deng L, Kline J, Frazier WA (2015). CD47 blockade triggers T cell-mediated destruction of immunogenic tumors. Nat Med.

[15] Bianchi ME, Mezzapelle R (2020). The chemokine receptor CXCR4 in cell proliferation and tissue regeneration. Front Immunol.

[16] Apetoh L, Ghiringhelli F, Tesniere A, Obeid M, Ortiz C, Criollo A (2007). Toll-like receptor 4-dependent contribution of the immune system to anticancer chemotherapy and radiotherapy. Nat Med.

[17] Yamazaki T, Hannani D, Poirier-Colame V, Ladoire S, Locher C, Sistigu A (2014). Defective immunogenic cell death of HMGB1-deficient tumors: compensatory therapy with TLR4 agonists. Cell Death Differ.

[18] Chow KYC, Brotin E, Khalifa YB, Carthagena L, Teissier S, Danckaert A (2010). A pivotal role for CXCL12 signaling in HPV-mediated transformation of keratinocytes: clues to understanding HPV-pathogenesis in WHIM syndrome. Cell Host Microbe.

[19] Nakamura N, Fujii M, Tsukahara T, Arai M, Ohashi T, Wakao H (1999). Human T-cell leukemia virus type 1 Tax protein induces the expression of STAT1 and STAT5 genes in T-cells. Oncogene.

